# Evaluation of Liver Grafts During Machine Perfusion at 20°C via Metabolization of 13C‐Methacetin

**DOI:** 10.1111/eci.70250

**Published:** 2026-08-13

**Authors:** Charlotte von Horn, Laura Malkus, Derya Berger, Stefanie Bertram, Thomas Minor

**Affiliations:** ^1^ Surgical Research Department University Hospital Essen Essen Germany; ^2^ Institute of Pathology University Hospital Essen Essen Germany

**Keywords:** liver evaluation, machine perfusion, methacetin

## Abstract

**Background:**

Evaluative measures, helping to decide whether a marginal graft can be used for transplantation or rather be discarded are of utmost importance but yet basically underdeveloped. Recent technical developments allow for the isolated detection of 13C‐labelled carbon dioxide in the oxygenator exhaust of an isolated perfused organ. By adding a 13C‐labelled tracer, its metabolism can be monitored by measuring the increase in 13C carbon in the exhaust air. Here we evaluated the potential of the technique to evaluate pig livers during machine perfusion.

**Methods:**

Porcine livers (six per group) were subjected to 5, 20 or 45 min of warm ischemia and cold stored overnight. Evaluative machine perfusion was done with MPS at 20°C for 2 h. After injection of 13C‐methacetin as a tracer, ^13^CO_2_ as metabolic end‐product can be quantified by high precision laser‐based spectroscopy in the gas outflow of the oxygenator.

**Results:**

The ^13^CO_2_ test showed clear differences between all three groups, and provided superior discriminative potential when compared to conventional parameters (e.g., flow, transaminases, lactate). ROC curve analysis for discrimination between 5 and 20 min of warm ischemia revealed an area under the curve of 0.916 while comparison of 20 and 45 min of warm ischemia results in an area under the curve of 1.0 (*p* < 0.01).

**Conclusion:**

Thus, the 13C turnover test appears to have the potential of an objective, quantitative and valuable adjunct in the toolbox for pre‐transplant evaluation of questionable liver grafts.

## Graphical abstract

Evaluative measures, helping to decide whether marginal grafts can be used for transplantation or rather be discarded are of utmost importance but yet basically underdeveloped. Livers of varying quality were subjected to machine perfusion at 20°C for 2 h. After injection of 13C‐methacetin as a tracer, ^13^CO_2_ as metabolic end‐product can be quantified. The ^13^CO_2_ test showed clear differences between all three groups and appears to have the potential for pre‐transplant evaluation of questionable liver grafts.

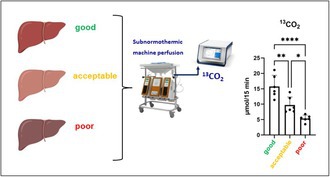

**Abbreviations**
ALT: alanine aminotransferase
AUC: area under the curve
HTK: histidine‐tryptophan‐ketoglutarate
LiMAx: liver maximum capacity
MP: machine perfusion
ROC: receiver operating characteristics
WI: warm ischemia

## 1 Introduction

Due to the increasing shortage of available donor organs, liver transplants with extended criteria are increasingly being accepted. However, the reduced tolerance of such organs to storage and transport outside the donor organism may negatively affect parenchymal function and result in limited or no functional recovery after transplantation.

To prevent overly optimistic use of non‐ideal organs that might result in an increased rate of primary dysfunction and early organ loss, most surgeons remain rather reluctant to accept critical organs. The resulting low utilization rate of donor livers of questionable quality [1] further contributes to the global organ shortage.

As efforts are made to expand the boundaries of organ acceptance criteria, evaluation criteria that help the surgeon decide whether a marginal organ should finally be considered for a promising transplantation or should rather be discarded are, however, becoming increasingly important [1].

In the past, a plethora of biomarkers have been studied to provide information on organ integrity during hypothermic or normothermic machine perfusion.

Sometimes a certain correlation, for example, of enzyme loss during normothermic machine perfusion, with the outcome after transplantation (again essentially determined by enzyme loss during reperfusion) [2] could be established. However, others often also showed no reliable correlation with regard to subsequent liver function [3, 4, 5], so that most biomarkers were found to be helpful but not accurate enough to carry a decision to reject or accept an organ [6].

Similarly, functional parameters such as lactate clearance or bile production could not be corroborated as reliable criteria to distinguish between livers with good or poor functional recovery after transplantation [7, 8].

Other methods, such as isolation of mitochondria from biopsy material followed by polarographic determination of mitochondrial respiratory characteristics [9], appear to correlate with a clinical outcome score after transplantation but are not immediately available in real time due to methodological constraints.

A worthwhile alternative has recently been demonstrated by transferring a clinical method of liver function measurement to the situation of the isolated perfused donor liver.

By measuring the proportion of the non‐radioactive 13C carbon isotope in exhaled carbon dioxide after prior intravenous administration of 13C‐labelled methacetin, the enzymatic performance of the cytochrome P450 1A2 system in the human organism can be estimated [10]. Since this enzyme system only occurs in the liver, this test procedure has been established clinically as the LiMax test for evaluating liver function for example, before planning partial liver resections, with the help of which it is possible to improve the assurance of sufficient residual function after removal of tumour‐bearing liver tissue [11].

A modification of this procedure for use under isolated machine perfusion of the liver is possible by injection of 13C‐labelled methacetin into the perfusate circuit and parallel detection of the resulting amounts of 13C carbon dioxide in the gas outlet of the oxygenator [4].

It is the aim of the present study to scrutinize the respective evaluative potential of measuring the enzymatic conversion of methacetin by non‐invasive spectroscopic measurement of 13C carbon isotopes during ex vivo machine perfusion of liver grafts.

To this purpose we have chosen a porcine liver preservation model that allows for the a priori definition of several experimental groups with different degrees of organ graft quality.

Using deliberately created ‘good’, ‘fair’ and ‘bad’ liver grafts, the 13C test shall be evaluated for its potential to discriminate between the groups after application of 13C methacetin. Moreover, its discriminative potential shall be compared to the use of yet available conventional biomarkers that will be determined during the same experiments.

## 2 Materials and Methods

### 2.1 Animals and Surgical Procedures

The study is reported in accordance to the recommendations in the ARRIVE guidelines (PLoS Bio 8 (6), e1000412, 2010).

All experiments were carried out on female domestic pigs (German Landrace × Pietrain), aged between 10 and 12 weeks. Animals were treated according to the rules and after approval of the responsible authorities (Landesamt für Natur, Umwelt und Klima—LANUK), NRW, Germany (AZ 81‐02.04.2023.A285). Federal law regulating the protection of animals and the principles of laboratory animal care (NIH publication vol 25, No 28, revised 1996) was followed. All experiments were carried out in accordance with the EU Directive 2010/63/EU for animal experiments.

The animals were kept at 22°C room temperature and received standard feed and water ad libitum during the adaptation period. After an acclimatization period of at least 10 days, the animals were used for the experiment. All animals were kept in pairs to allow social contact during the housing period.

### 2.2 Liver Retrieval

Sedation of the animal was achieved by intramuscular injection of tiletamin/zolazepam (4 mg/kg). General anaesthesia was induced after canulation of the ear vein by intravenous injection of propofol (3 mg/kg) followed by intubation and mechanical ventilation with isoflurane (1.5%–2%) in air/oxygen. A continuous infusion of fentanyl (0.015 mg/kg/h) was started to deepen analgesia.

An incision in the animal's neck exposed the internal jugular vein and a large‐lumen indwelling venous catheter was inserted and used to continue administration of fluids as well as analgesic medication during the ongoing operation.

The abdomen was opened via a median laparotomy. The liver was then released from its ligamentous structures. The bile duct was visualized and cannulated with a catheter. Hereafter, the vascular structures, the portal vein, the hepatic artery, the infra‐ and supra‐hepatic vena cava are visualized and isolated.

After completion of surgical preparation of the liver, heparin (25,000 IE) was given via the jugular catheter.

At the conclusion of the operation, cardiac arrest was induced by injection of 60 mL of potassium chloride (7.45%) via the jugular catheter. The portal vein was cannulated 5, 20 or 45 min thereafter with a large lumen catheter and 4°C cold preservation solution was infused into the liver. Opening of the superior vena cava allowed for good flow of the preservation fluid through the liver. It followed the transection of the vessels as far from the liver as possible and storage of the isolated liver in 4°C cold preservation solution. Eventually, isolated perfusion of the hepatic artery was performed with 100 mL of Custodiol (HTK) preservation solution.

### 2.3 Study Design

Three groups of livers with graduated injury prior to organ retrieval were tested by functional evaluation through subnormothermic machine perfusion.

Livers were retrieved 5 min (Group 1; *n* = 6), 20 min (Group 2; *n* = 6) or 45 min (Group 3; *n* = 6) after induction of cardiac arrest in the donor animal and rinsed with commercial HTK preservation solution. After 16 h of cold storage in HTK solution (2°C–4°C), connection to subnormothermic machine perfusion was performed for evaluation (see below for details).

### 2.4 Evaluative Machine Perfusion

After 16 h of static storage, livers from all groups were put on a machine perfusion circuit and subjected to an end‐ischemic machine perfusion for 2 h.

The livers were placed in a closed perfusion chamber that also serves as a reservoir for the perfusate. According to our previous experiences [12, 13], the temperature of this chamber with the perfusate is controlled by a circulating thermostat and gradually increased from 8°C up to 20°C during the first 30 min and then kept constant for the remaining period. Perfusion of the liver is dual via both the portal vein and the hepatic artery in order to represent the physiological processes. The perfusion medium is drained through the vena cava and made available in the reservoir for recirculation.

The portal perfusion is controlled by means of a blood pump (Biopump, Fa. Medtronic). An in‐line ultrasound transit time sensor is integrated into the tubing system to monitor the current flow rates at any given time, which is read out via a connection to the Medtronic bioconsole. Portal perfusion pressure is set to 4 mmHg and continuously monitored.

The arterial line is perfused with a pressure of 40 mmHg, for which a second pump is used.

The arterial perfusion pressure is measured by a TAM‐A Plugsys pressure amplifier with probe, which is connected to the arterial tubing system via a Luer adapter. A servo‐controlled feedback loop controls the pump supplying the arterial limb in such a way that a constant pressure is applied to the a. hepatica (40 mmHg). An oxygenator connected into the circuit is used to adequately oxygenate the circulating perfusate. Perfusate samples are taken and the oxygen content is measured using an acid base laboratory (ABL 800, Radiometer, Copenhagen). At the same time, this measurement also allows control of the perfusate pH and CO_2_ content.

### 2.5 Evaluation of Liver Integrity

During the 2nd period of end‐ischemic machine perfusion at constantly 20°C, metabolic measurements were performed evaluating the throughput of 13C methacetin as a tracer by the cytochrome P450 1A2 system of the liver.

The principle of this test is similar to the clinically established LiMAx test that has been validated for the evaluation of liver function prior to hepatic surgery [11].

The present study was aimed to transfer this test to the setting of an isolated organ, perfused significantly below normothermia at 20°C and using the novel high precision 13CORlab device (ArgosMED GmbH, Karlsruhe Germany).

The exhaled gas from the oxygenator is directed through the device and direct spectroscopic detection of ^13^CO_2_ as well as ^12^CO_2_ is possible during gas transit through the detector [14]. Baseline values for ^13^CO_2_ and the ^13^CO_2_/^12^CO_2_ ratio are recorded before application of the tracer. After injection of a bolus of 13C methacetin (20 mg) into the perfusion circuit metabolic turnover of 13C methacetin can be followed by determination of the molecular amount of ^13^CO_2_ that is released from the oxygenator on top of the previously registered baseline level [14]. The device is able to simultaneously record flow, pressure and temperature of the gas during its passage through the detector. This allows for the calculation of the exact amount of substance of ^13^CO_2_ at a given time point with a sampling rate of 1/s. By multiplying the metabolized concentration of ^13^CO_2_ with the total volume per time divided by the molar gas volume at standard conditions the amount of substance can be determined. The amount of substance detected during the first 15 min after injection of the tracer is taken as a readout of the respective metabolic activity of the liver graft.

### 2.6 Conventional Parameters to Evaluate Liver Integrity

Enzyme loss into the perfusate was examined as an indicator of parenchymal damage. For this purpose, the enzyme levels of alanine aminotransferase (ALT) [2] were determined photometrically using commercial standard methods.

As suboptimal clearance of lactic acid has recently been reported as common feature of livers, developing primary nonfunction [15], perfusate levels of lactate had been determined using an acid base laboratory (ABL 800, Radiometer, Copenhagen, DK).

Changes in vascular conductivity were monitored by continuous measurement of portal venous and arterial flow upon pressure‐controlled perfusion.

### 2.7 Histology

Liver tissue was collected at the conclusion of the experiments, embedded in paraffin and 3‐μm‐thick sections were stained with haematoxylin and eosin. A pathologist, blinded as to group identity, rated the extent of sinusoidal congestion, central ballooning, and necrosis on a scale from 0 to 4 according to the Suzuki classification [16].

### 2.8 Details of Biometric/Statistical Planning

The primary endpoint for planning the necessary number of cases per group is the discrimination between groups of livers with factually different quality by changes in metabolism of methacetin during ex vivo machine perfusion.

Based on previous studies in the kidney analysing the turnover of acetate with a similar technique [14] we expected a standard deviation of 33% and a minimum inter‐group difference of 75%. These specifications result in a necessary number of trials per group of *n* = 6 for the post hoc test after multi‐group comparison with univariate ANOVA and pairwise Bonferroni correction, with a 5% probability of error in the first (alpha) order and a power of 80% (BiAS windows, 11.12, epsilon).

All values were expressed as means ± SD unless otherwise indicated. Differences between groups were tested by one‐way ANOVA and parametric comparison of the means using Tukey–Kramer test. Statistical significance was set at *p* < 0.05. ROC curve data were calculated using Prism 9.01 (GraphPad Software Inc., San Diego, CA, USA). ROC curve analyses were performed after successful testing for normal Gaussian distribution by the method of Kolmogorov and Smirnov using Prism 9.01 (GraphPad Software Inc., San Diego, CA, USA).

## 3 Results

The livers used in this study were retrieved from 18 pigs, ageing between 10 and 12 weeks, and randomly subjected to 5, 20 or 45 min of warm ischemia prior to cold preservation in order to represent organ grafts of good, moderate and poor quality. Organ weight averaged 692 ± 66 g vs. 726 ± 76 g vs. 731 ± 56 g, respectively, with no significant differences between the groups.

### 3.1 Methacetin Metabolization Upon Subnormothermic Machine Perfusion

Stable values for ^13^CO_2_ and ^12^CO_2_ at the oxygenator exhaust could be recorded upon machine perfusion within 30 min after reaching final perfusion temperature of 20°C.

**FIGURE 1:**
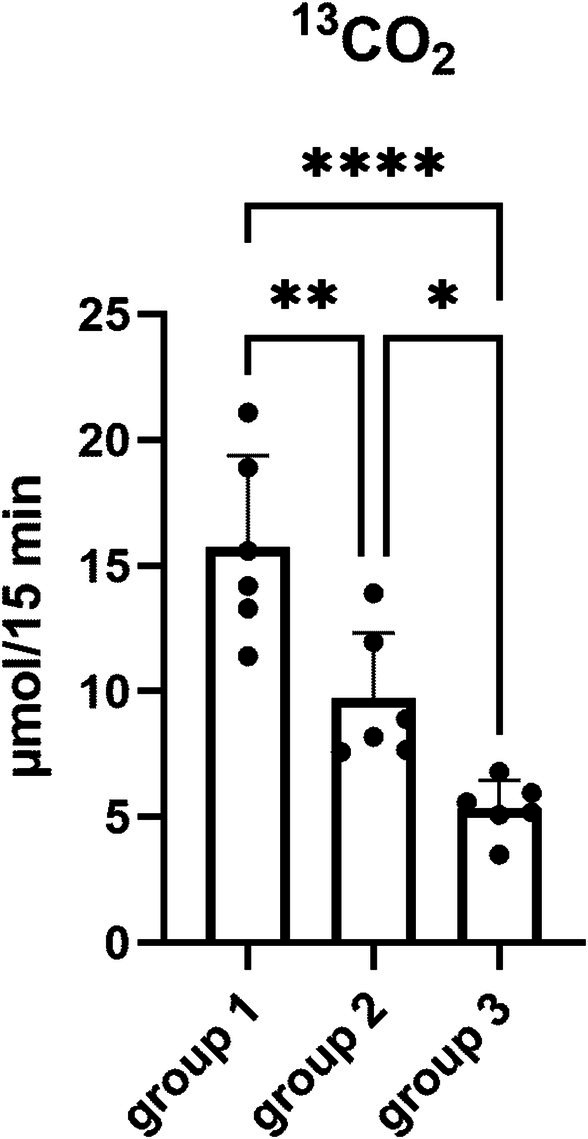
Differences between 13C turnover of liver grafts obtained during machine perfusion. Data are given a mean ± SD and individual values of *n* = 6 per group; *, **, ****p < 0.05, 0.01, 0.0001, respectively, by one‐way ANOVA and Tukey–Kramer test.

After injection of the tracer a swift increase of the ^13^CO_2_ signal was observed at the detector in all of the groups. However, the slope of the ^13^CO_2_ signal was sensibly affected by the degree of liver damage incurred prior to preservation (cf. Figure 1). Thus, turnover rates of methacetin were significantly lower in Group 3 than in Group 2 while the turnover rates of Group 2 were significantly surpassed by the livers of Group 1.

### 3.2 Conventional Parameters Upon Ex Vivo Machine Perfusion

**FIGURE 2:**
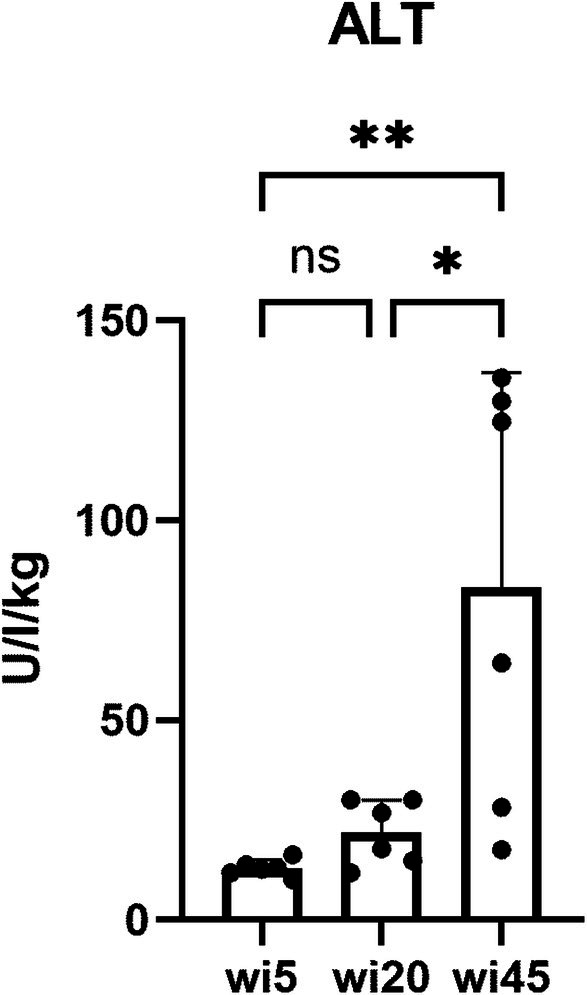
Perfusate levels of alanine aminotransferase (ALT) at the end of machine perfusion at 20°C. Data are given a mean ± SD and individual values of *n* = 6 per group. Differences among the groups were tested by one analysis of variances and post hoc testing with the Tukey Kramer test; *p < 0.05 **p < 0.01.

Perfusate levels of the alanine aminotransferase (ALT) at the end of the machine perfusion period were analysed as one of the most common read‐outs of morphological liver injury. It was seen that hepatic release of ALT fairly well responded to the degree of liver injury albeit no significant differences could be evidenced between Group 1 and Group 2, that is, the two groups of lesser injury (Figure 2). Although the more severe ischemic challenge in Group 3 resulted in a significant increase of mean ALT perfusate values, individual results showed a huge variability that would have precluded the discrimination from the groups of lesser injury in two cases.

**FIGURE 3:**
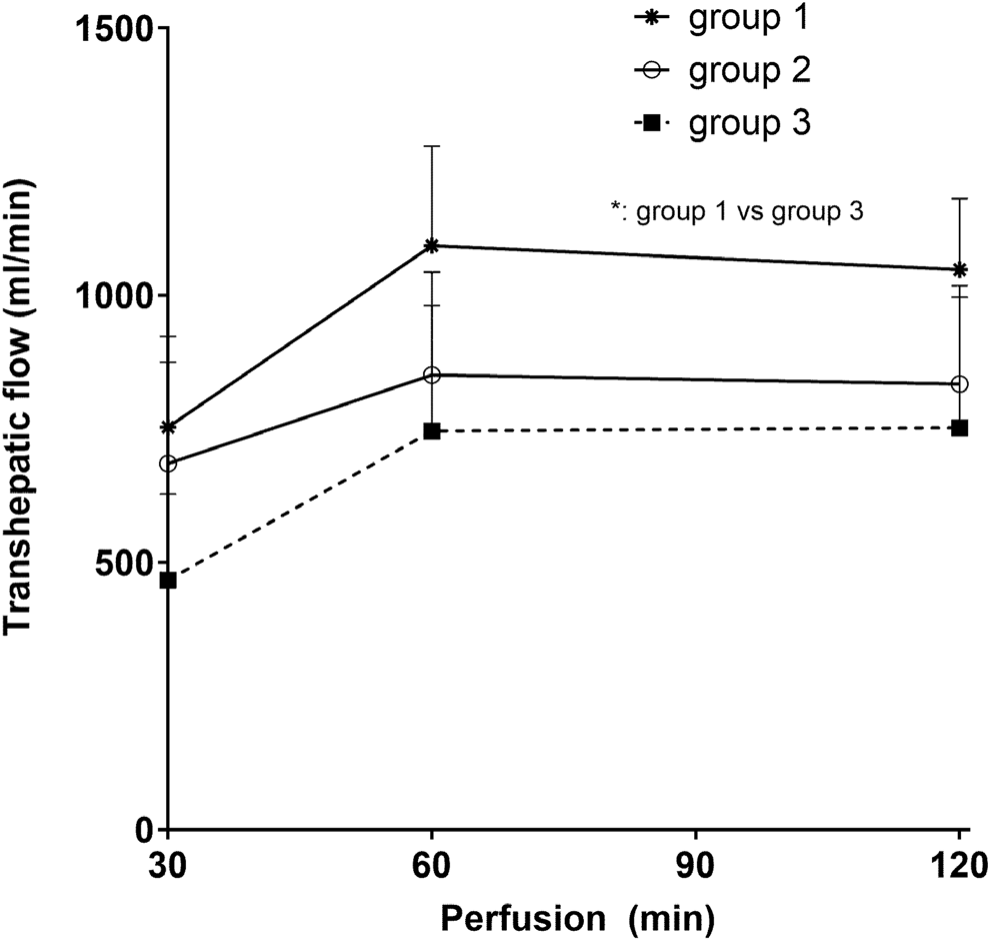
Transhepatic flow during machine perfusion at 20°C. Data are given as means ± SD of *n* = 6 experiments per group. Differences among the groups were tested by comparison of the areas under the curve using analysis of variances and post hoc testing with the Tukey Kramer test; *p < 0.05.

Total transhepatic flow during pressure constant machine perfusion is depicted in Figure 3. Although the flow values in Group 1 were clearly superior to both other groups, no difference could be substantiated between Groups 2 and 3.

**FIGURE 4:**
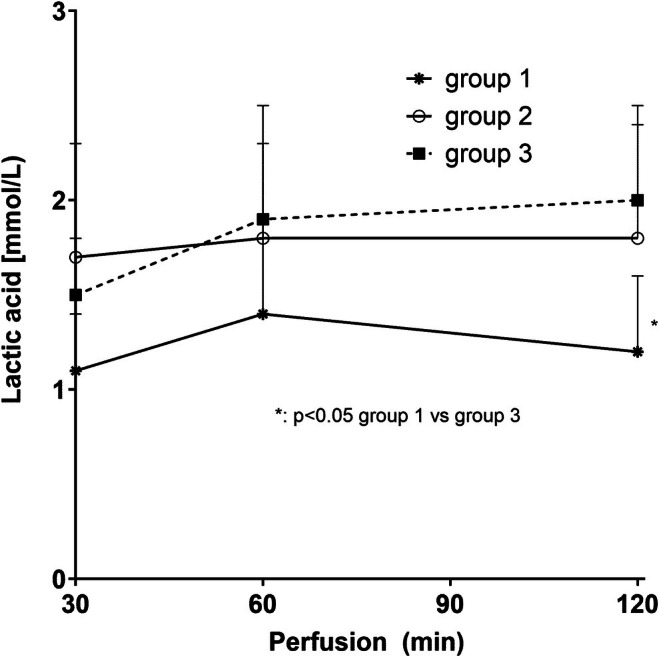
Perfusate levels of lactic acid during machine perfusion at 20°C. Data are given as means ± SD of *n* = 6 experiments per group. Differences among the groups were tested by comparison of the areas under the curve using analysis of variances and post hoc testing with the Tukey Kramer test; *p < 0.05.

Lactate accumulation over time revealed similar results. While tendentially lower values of lactic acid in the perfusate could be delineated in Group 1, that significantly differed from those in Group 3, no significant differences were seen between Groups 2 and 3 (cf. Figure 4).

Histological examination revealed rising injury score levels along with the extension of warm ischemic injury, mainly due to increased prominence of tubular dilatation and cellular oedema in the more severely damaged groups. Mean score values in Group 1 (1.05 ± 0.39) differed significantly from those in Group 2 (1.61 ± 0.33; *p* = 0.05) and Group 3 (1.84 ± 0.18; *p* < 0.01). The difference between the two latter groups did not reach statistical significance.

### 3.3 Receiver Operator Characteristics

**FIGURE 5:**
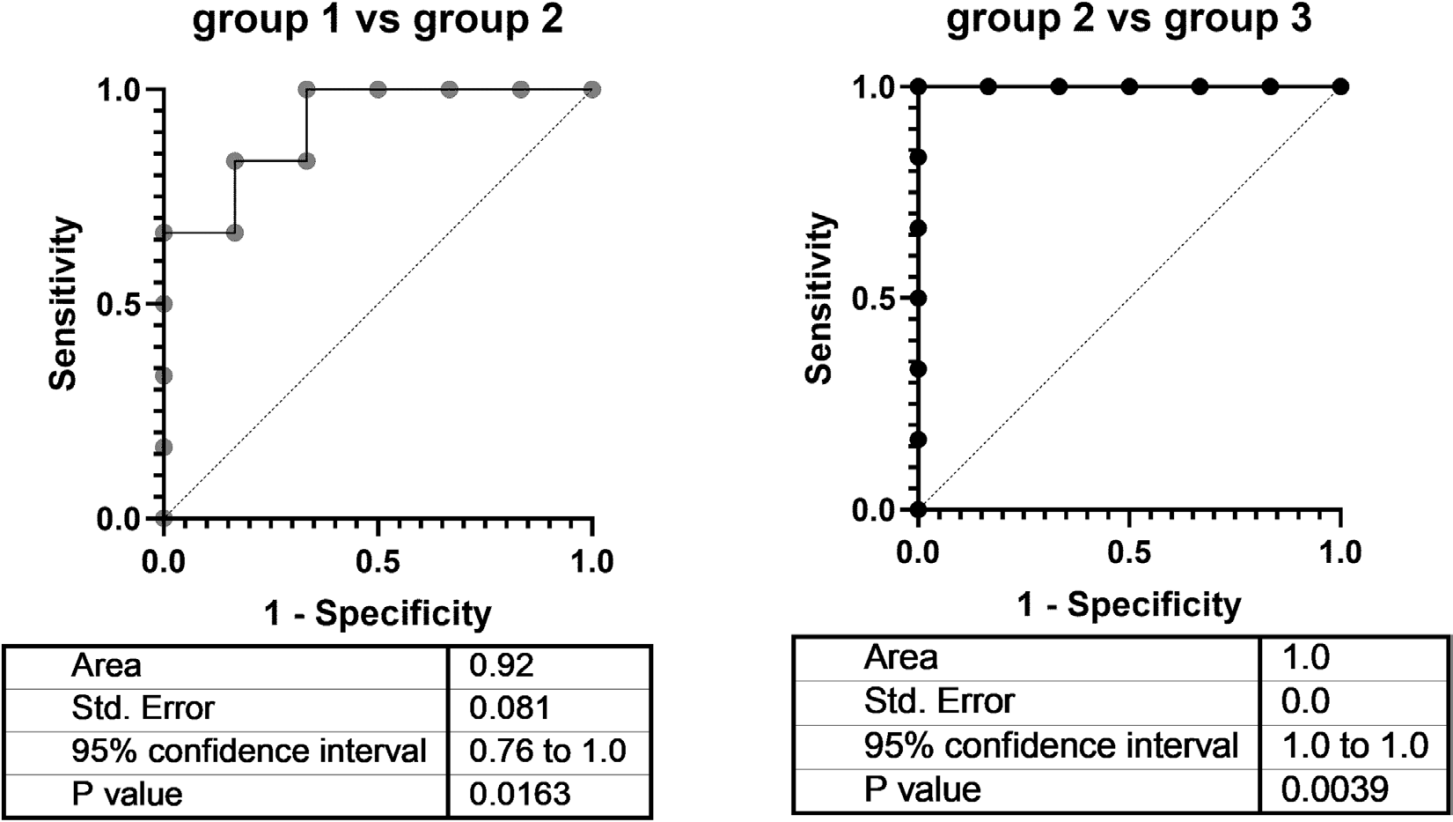
Receiver‐operating characteristics curves illustrating the accuracy for 13C‐methacetin metabolization observed during machine perfusion to allow for discrimination between livers of different quality. Data were calculated from *n* = 6 per group.

A pivotal goal of the present study was to disclose sensitivity and specificity of the methacetin turnover test to discriminate between the predefined levels of liver quality. To this purpose, receiver operating curves (ROC) were established defining the area under the curve for comparison of accurate inter‐group discrimination by 13C measurements (cf. Figure 5). In these analyses, the area under the curve consistently reached values above 0.9, irrespective if the comparison was made between Group 1 and Group 2 or between Group 2 and Group 3. Thus, the methacetin turnover, as evaluated by 13C output, proved to be significantly discriminative between mild and moderate as well as moderate and severe liver injury.

By comparison, perfusate levels of ALT only allowed for discrimination between Group 1 and 2 (area under the curve [AUC] = 1.0; *p* < 0.001) but failed to yield significant results upon comparison of Group 2 and 3 (AUC = 0.83; *p* = 0.055; cf. Table S1).

**FIGURE 6:**
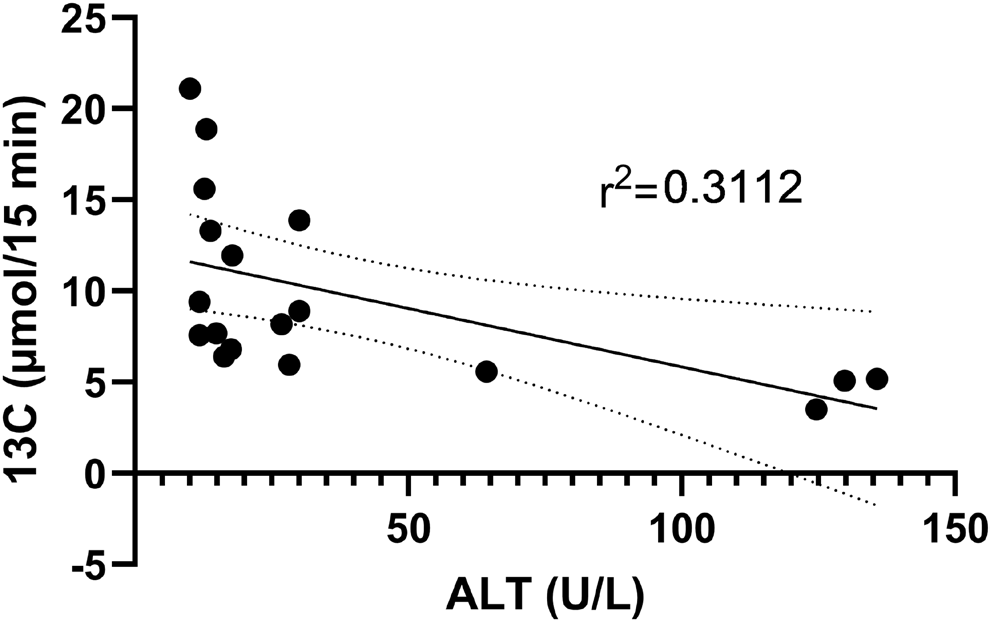
Correlation of methacetin turnover rates with maximal perfusate levels of ALT as morphological injury marker.

At last, we were looking for a correlation between the functional data of the methacetin turnover test and the perfusate levels of ALT as a mere morphological injury marker. As depicted in Figure 6, we could disclose a significant, albeit rather weak (Pearson correlation coefficient (*r*) = −0.5579; 95% CI: −0.8130 to −0.1230) correlation between the two parameters.

## 4 Discussion

The advent of machine perfusion techniques for preservation or reconditioning of liver grafts has brought up the option of assessing graft quality using perfusate biomarkers. In addition to relying on subjective evaluation of graft appearance or medical history data, measuring discrete parameters obtained while the liver is on the pump would allow for a more objective assessment of organ suitability for transplantation [17].

However, the analysis of specific morphological injury markers is often rather time‐consuming and not available as a point of care procedure.

Moreover, biomarkers were often found to be helpful, but not accurate enough to carry a decision to reject or accept an organ [6]. Functional assessment of the livers might hence be a more expedient approach than mere determination of graft injury [18]. Evaluation of liver function has recently been proposed by isolation of mitochondria from biopsy material, followed by high‐resolution respirometry for determination of mitochondrial respiratory characteristics [9]. Although the results of this method appear to correlate with a clinical outcome score after transplantation, it is a technically advanced and time‐consuming procedure that may not always meet the need for an immediately available assessment tool. Moreover, biopsy related evaluation methods are dependent on the representativeness of the tissue sample while integral assessment of the whole organ is not. Since in liver transplantation the survival of the patient depends on a functional liver, the prediction of sufficient organ function after transplantation by a reliable method is of particular importance. Schurink et al. have recently shown that the clinically validated liver maximum capacity (LiMAx) test, that is, measurement of methacetin turnover after injection of a 13C labelled tracer substance by detection of production of free ^13^CO_2_, can also be applied to the isolated liver during normothermic machine perfusion [4].

However, machine perfusion at normothermia is a rather complex procedure and the putative need of erythrocytes as oxygen carriers has yet complicated its translation into clinical practice [19]. Additional drawbacks might be seen in the increased risk of microbial contamination and the acute challenge of warm ischemic injury if the device fails inadvertently [20]. Subnormothermic machine perfusion has thus been advocated as possible alternative with promising results in mitigating reperfusion injury comparable to normothermic machine perfusion [21, 22]. Unfortunately, viability assessment below normothermic temperatures is not currently well defined [23, 24].

In this study we therefore evaluated the diagnostic potential of the ^13^CO_2_ test at 20°C in a rather uncomplicated machine perfusion set‐up.

It was seen that the analysis of ^13^CO_2_ at the CORLab detector allowed for a pretty well discrimination of different metabolic states as associated with different degrees of ischemic tissue injury. The evaluation of the cytochrome P450 1A2 system has been established clinically for evaluating liver function since this enzyme system only occurs in the liver. Under the conditions of an isolated organ, however, one is no longer dependent on investigating a specific metabolic pathway present only in the target organ, since interference by metabolic processes of other organs is methodically excluded.

For instance, the metabolic activity of the citrate cycle can be investigated by detecting the metabolism of added 13C‐labelled acetate during ex vivo machine perfusion as has already been shown in the kidney [25]. In our model, the use of 13C acetate as tracer disclosed results quite similar to the methacetin‐test, producing a significant difference between lives of Group 3 and Group 2 (20.5 ± 9.9 vs. 41.1 ± 14.9 μmol/30 min, *p* < 0.05). Other metabolic pathways may also be analysed by this technique, for example, long chain fatty acids as energy sources during mitochondrial β‐oxidation reactions [26].

The extent of liver injury in the respective groups was chosen according to the empirical likelihood of liver dysfunction after porcine transplantation. Clinical recommendations for acceptance of livers after observed cardiac arrest assume a maximum tolerable time of circulatory arrest of 30 min [27]. In pig liver transplantation, the critical time of tolerable warm ischemia seems to lie in a comparable range, since after 15 min a functional recovery ensuring survival can still be observed, which, however, decreases significantly after 30 min of warm ischemia [28, 29]. Removal after 45 min of cardiac arrest in the donor animal is associated with a low probability of ever resuming adequate function after transplantation [30, 31].

Based on this, the discrimination of Groups 2 and 3 should be of primary importance for a liver integrity test since it separates the presumably failing liver grafts from those of marginal but sufficient quality for live supporting function.

Interestingly, functional data from the ^13^CO_2_ test were only roughly linked to the morphological injury readout (ALT). This may point to a differential impact of the ischemic challenge on functional or morphological tissue alteration, respectively.

Although both parameters seem to concordantly respond to the prolonged ischemic injury of the graft, they apparently refer to entities of injury that are not neatly deductible one from another. Given that clinical practice of normothermic perfusion is evolving toward longer periods of time (for logistical as well as reconditioning purposes) the test could also serve as a tool for monitoring the success of treatment or intervention. Repetition of the measurement is easily possible after several hours, as soon as the initial tracer substance is fully metabolized. In our hands, replicate measurements on a healthy liver gave a readout of 21.3 μmol/15 min after 30 min of machine perfusion and 20.9 μmol/15 min after 240 min of machine perfusion.

Limitations of our study relate to the isolated ex vivo perfusion model, which only partly reflects the actual situation in vivo.

In our study we have focussed on the discriminative power of the ^13^CO_2_ test using a model with highly defined different degrees of liver alteration. Although this model does not exactly mirror the multitude of clinical reasons for reduced liver function it is the only reproducible experimental approach in pigs to disclose different states of parenchymal injury and intended to allow for a primary validation of the new screening method. A drawback of this model lies in the relatively small number of replicates per group and the fact that the operators were not blinded.

Our approach also contrasts with the evaluation of previous multimodal clinical assessment criteria that were used upon poor but not empirically predefined liver grafts and validated by correlation with ulterior recovery of the grafts after transplantation. Our data cannot directly be compared these clinical scores.

Although bile flow could not appropriately be addressed in our model at 20°C [32], most of the parameters looked for by established viability testing protocols (e.g., flow values, lactate, transaminases) [33, 34, 35] have also been evaluated in our model as single value and turned out to provide inferior discrimination of the different groups than the ^13^CO_2_ test. Final validation of this tool has, of course, to be done upon long term follow up in clinical exploration, also including grafts suffering from reduced resilience due to reasons other than ischemic injury like steatosis, inflammation, higher age or accompanying diseases of the donor. Due to species‐specific CYP1A2 differences [36], cut off values for methacetin turnover rates will anyhow have to be established by post‐transplant recovery of human grafts in clinical routine.

Ease of use as well as real time availability of the non‐invasive methacetin‐turnover test argue in favour of its prospective clinical testing as it might become a useful additional adjunct for deciding whether to use or to discard an individual donor graft.

## Author Contributions

C.v.H.: participated in research design, writing of the paper and data analysis. L.M.: participated in performance of the research. D.B.: participated in performance of the research. S.B.: participated in data analysis. T.M.: participated in research design, writing of the paper and data analysis.

## Funding

The authors have nothing to report.

## Ethics Statement

Animals were treated according to the rules and after approval of the responsible authorities (Landesamt für Natur, Umwelt und Klima ‐LANUK), NRW, Germany (AZ 81‐02.04.2023.A285). Federal law regulating the protection of animals and the principles of laboratory animal care (NIH publication vol 25, no 28, revised 1996) was followed. All experiments were carried out in accordance with the EU Directive 2010/63/EU for animal experiments.

## Conflicts of Interest

The authors declare no conflicts of interest.

## Supporting information


**Table S1:** Area under the receiver operator curve (ROC), 95% confidence interval (CI), sensitivity, specificity and level of significance to discriminate between livers from Group 2 and from Group 3 by production of ^13^CO_2_ after injection of ^13^C‐methacetin or ALT activities after 2 h of machine perfusion.

## Data Availability

Data that support the findings of this study are available from the corresponding author upon reasonable request.
