# Supplementary material for: Evaluation of Liver Grafts During Machine Perfusion at 20°C via Metabolization of 13C‐Methacetin
Source: Eur J Clin Invest. 2026 Aug 13;56(8):e70250. doi: 10.1111/eci.70250 (PMC13473386; doi:10.1111/eci.70250)
Supplement: Supplementary file 1 [file ECI-56-e70250-s001.docx]

Suppl. Table 1: Area under the receiver operator curve (ROC), 95% confidence interval (CI), sensitivity, specificity and level of significance to discriminate between livers from group 2 and from group 3 by production of ^13^CO_2_ after injection of ^13^C-methacetin or ALT activities after 2h of machine perfusion.

Area(95% CI)	cut off	sensitivity(95% CI)	specificity(95% CI)	P=

^13^C-acetate	1.0	0.61-1.0	7.2	1.0 (0.61-1.0)	1.0 (0.61-1.0)	0.004

ALT	0.83 (0.59-1.0)	28	0.67 (0.30-0.94)	0.83 (0.44-0.99)	0.055
